# Review: Application and Prospective Discussion of Machine Learning for the Management of Dairy Farms

**DOI:** 10.3390/ani10091690

**Published:** 2020-09-18

**Authors:** Marianne Cockburn

**Affiliations:** Agroscope, Competitiveness and System Evaluation, 8356 Ettenhausen, Switzerland; marianne.cockburn@agroscope.admin.ch

**Keywords:** sensor, cluster, data analysis, big data, data integration, smart farming

## Abstract

**Simple Summary:**

Machine learning (ML) offers new approaches for analyzing data and is particularly interesting for large datasets. Dairy farmers implement a wide range of sensors, which create large amounts of data, in farming. Therefore, they offer an interesting area for data-driven research. In this review, we show how ML methods have already been used in the scientific literature and describe the potential that these may offer for the future. We found that ML methods were applied to predict data in a variety of areas in dairy farming such as milk yield or energy consumption; however, larger integrated datasets are required to improve the reliability of the algorithms developed.

**Abstract:**

Dairy farmers use herd management systems, behavioral sensors, feeding lists, breeding schedules, and health records to document herd characteristics. Consequently, large amounts of dairy data are becoming available. However, a lack of data integration makes it difficult for farmers to analyze the data on their dairy farm, which indicates that these data are currently not being used to their full potential. Hence, multiple issues in dairy farming such as low longevity, poor performance, and health issues remain. We aimed to evaluate whether machine learning (ML) methods can solve some of these existing issues in dairy farming. This review summarizes peer-reviewed ML papers published in the dairy sector between 2015 and 2020. Ultimately, 97 papers from the subdomains of management, physiology, reproduction, behavior analysis, and feeding were considered in this review. The results confirm that ML algorithms have become common tools in most areas of dairy research, particularly to predict data. Despite the quantity of research available, most tested algorithms have not performed sufficiently for a reliable implementation in practice. This may be due to poor training data. The availability of data resources from multiple farms covering longer periods would be useful to improve prediction accuracies. In conclusion, ML is a promising tool in dairy research, which could be used to develop and improve decision support for farmers. As the cow is a multifactorial system, ML algorithms could analyze integrated data sources that describe and ultimately allow managing cows according to all relevant influencing factors. However, both the integration of multiple data sources and the obtainability of public data currently remain challenging.

## 1. Introduction

**Figure 1:**
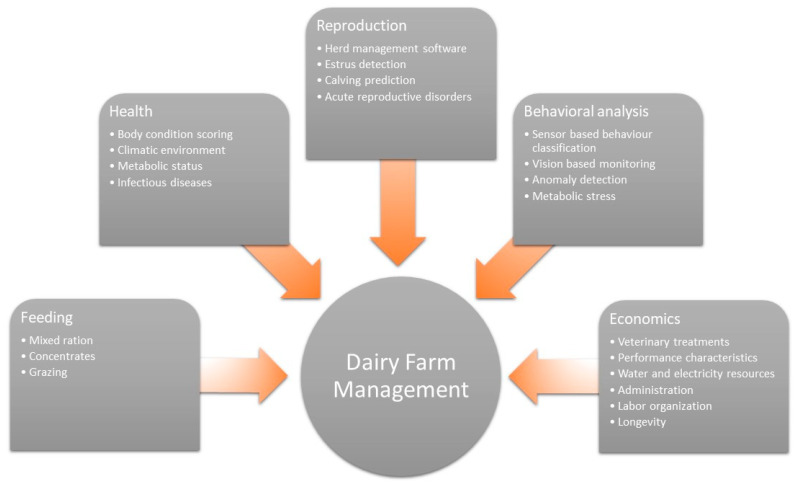
Systematic structure of dairy farm management.

Economic pressure requires increased efficiency in dairy production, which has come along with high-yielding dairy cows, large herds, and a strong movement toward loose housing systems. Consequently, improving animal welfare on the farm can amplify profits, as it has the potential to reduce costs related to healthcare and poor yields, and as such, improve the sustainability and efficiency of dairying [1]. Only well-managed animals will meet the required production levels, while unhealthy animals will need early culling, and thus they will exhibit decreased longevity, not be as profitable, and will accordingly be less sustainable [1,2]. Bell et al. [2] reported that 59% of Holstein Friesian cows are culled before their fourth lactation. Culling is often the result of poor health, where the main risk factors for culling are assisted calving, abortion and/or mastitis, higher age, fewer days in milk, and poor conception rates [2]. It has further been reported that 55% of lactations are associated with lameness-related health disorders, and 15% with mastitis or uterine infections [2]. This shows that, despite advanced knowledge concerning the management of dairy cows, many unsolved issues remain. Ensuring a healthy life for each individual cow has become a multifactorial challenge, which is difficult to manage under current conditions, particularly because reproduction, feeding, milking, and health aspects are considered separately (Figure 1). Novel methods in data analysis may now offer new approaches to aid synthesizing the systematic structure of dairy farms and so boost future management decisions.

Agricultural production data are widely available, but they are not used enough to inform on production-relevant tasks. To date, we can only estimate their potential, and thus utilizing these data is challenging. Therefore, only a few farmers recognize data management as a chance to improve their business. In human medicine, where the potential of machine learning (ML) algorithms has been recognized, the application of these techniques has improved diagnostics in a number of diseases such as heart disease, diabetes, dengue fever, and hepatitis [3]. Machine learning models such as random forest can hold categorical data and are insensitive to missing values. Furthermore, they have the power to analyze large datasets, which often are difficult to evaluate with traditional statistical models [3]. This highlights the prospects that ML techniques could offer for dairy farming. Analyzing large integrated datasets may allow providing farmers with better decision support systems, and as such, assist them to increase the wellbeing and efficiency of their animals.

Advances in sensing technologies have led to an increased availability of sensors in farming [4]. Milking machines, which deliver daily milk yield data, are the most commonly implemented sensors in dairy farming. Systems that monitor individual animal behavior, such as rumination, estrus, location, or rumen pH, are also becoming available [5,6,7]. Furthermore, many farmers’ record data on the cows’ individual concentrate feeding behavior. To date, however, only a few Swiss farmers use such precision technologies in practice, whereas higher adoption rates can be found in Australia, where particularly farms with larger herds implemented more technology [8,9]. Electronic cow identification and herd management software were the technologies with the highest adoption rates in Switzerland [8]. In the future, farmers expected the largest adoption of technologies in automatic sorting gates and estrus detection (72.9 and 76.4%), respectively [8]. In addition to these automatically collected sensor data, milk testing is conducted, and feed advisors regularly perform laboratory analysis on feed composition. Furthermore, weather stations provide continuous data on climatic conditions. The farmer typically stores and processes some of these data in a feeding and herd management software, but it is difficult to jointly analyze the different data.

ML algorithms, among other methods, present an approach to analyzing these datasets, which are increasingly becoming available on many farms. Machine learning is a subfield of artificial intelligence [10]. According to Liu [11], ML intends to effectively reproduce human learning behavior, allowing for the automatic detection and acquisition of new information. The algorithms are hereby capable of detecting clusters in large datasets with numerous variables, predicting the onset of events, and learning from the data provided [12]. These ML approaches fall into the categories of supervised, unsupervised, and semi-supervised learning. Supervised learning requires labeling data and a training dataset, whereas unsupervised learning evaluates the data independently without labeling or a clear target variable; furthermore, semi-supervised learning approaches use datasets with only a small proportion of labeled data [12]. To date, new tools, and more specifically statistical, packages to process data have become available, making it easier to analyze data with artificial intelligence. While traditional statistical models evaluate data according to a certain theory, in ML approaches, the evaluation is data driven [13]. Therefore, some statisticians suggest that ML algorithms will usually provide a better result because they learn from the data provided, whereas traditional analysis methods are biased by the researcher’s hypothesis [13].

The current review aims to evaluate how ML approaches can promote the processing of on-farm sensor data to develop better decision support for farmers and ultimately improve their management.

## 2. Materials and Methods

A literature search covering the period between January 2015 and June 2020 was performed to create an overview of ML-related studies published in the dairy sector over the past five years. Citing additional literature was permitted if it appeared relevant to the field and indicated the potential of ML algorithms. The literature search was performed using Google Scholar and Scopus; however, due to its advanced search options, the analysis focused on Scopus searches. The Scopus search was: “(TITLE-ABS-KEY/ (“Search String”) AND PUBYEAR > 2014 AND (LIMIT-TO (DOCTYPE, “ar”) OR LIMIT-TO (DOCTYPE, “re”)).” See Table 1 for search strings.

Inclusion criteria were defined as peer-reviewed articles and review papers published in English. Due to the high number of search results, only studies in which the search string was found either in the “Title” or in the “Abstract, Title, or Key Words” were evaluated. Exclusion criteria comprised conference proceedings and studies in languages other than English. While analyzing relevant papers, the snowball method was used, and significant cited literature was included [14]. All studies were screened for their relevance to the field and stored in the referencing software Endnote X8 (Clarivate Analytics, Philadelphia, PA, USA). The relevance was defined as cow-related studies concerning dairy science or agriculture, whereas studies concerning dairy products or other related topics were considered as irrelevant. If the paper appeared valuable to the field by judgment of the author, it was read in full and important aspects were highlighted. While the search results considered only those studies that used or discussed ML as their main methodology, non-ML studies were additionally cited in order to put the ML studies in a context. In total, 97 papers were included in the results and discussion section of this review.

## 3. Results and Discussion

The current review demonstrates the significance of ML in agriculture by finding 869 articles mentioning the search string “machine learning agriculture” and 109 containing “machine learning dairy” in their abstract, keywords, or title. The number of search results for our search strings reflect the broad interest in the field of ML by the scientific community (Table 1). However, finding and analyzing the most meaningful results within the search strings was difficult. We found 101 search results that pointed to ML-related data evaluation methods in their title; of these, 84 papers concerned dairy science (Table 1). There was considerable overlap of search results for some search strings, as they presented subfields of other search strings.

**Figure 2:**
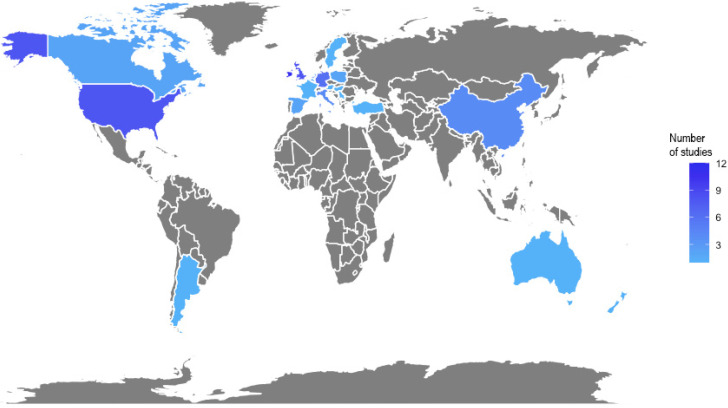
Origin of data used in the studies identified in the systematic literature search on machine learning in dairy science. Only original research considered (no reviews).

Despite these results, finding and reviewing the most relevant papers is problematic because numerous ML methods do not require mentioning these keywords in the document. In fact, it may even be possible that the application of specific and relevant methods is less likely to be mentioned under the search string “machine learning,” whereas less specific studies would be more likely to use this general term. This makes it difficult to find and analyze the most relevant literature. This has similarly been reported for a review on Big Data, which pointed out that, within the 613 peer-reviewed articles that resulted from their Big Data-related search strings, only 20 were considered most relevant and 94 were considered relevant to the topic, while the others only had little significance [15]. In the current review, we accounted for this issue by including search strings addressing the most popular ML methods. Although this strategy resulted in finding additional publications, we cannot conclude that it enabled us to find all relevant papers, as there are too many methods that can be referred to differently in each study. Within the search results, it became evident that most method-related hits appeared for the ML method “cluster*” (Table 1). This could be because various clustering methods employ the word “cluster”, while other methods do not use similar wordings. Therefore, the high number of search results containing the word “cluster*” should not be overrated. Due to the large number of articles published between 2015 and 2020, we were only able to screen articles in which the title, abstract, or keywords contained the search strings. In total, 97 articles were considered in this review. The most research studies were performed on Irish and American data (Figure 2).

Ultimately, it feels necessary to mention that ML methods are no more than an alternative approach for data evaluation. Thus, many articles will feel no need to point out the general term ML in the title of their paper, whereas authors would normally mention the specific method of data evaluation in the abstract. Thus, we acknowledge that we may be missing relevant literature that did not fall into the search strings, but we are confident that we can give an insightful outlook on current ML applications in dairy science.

### 3.1. General Findings and Outlook 

Data models have a great potential for agricultural farms. However, O’Grady and O’Hare [16] evaluated the availability and implementation of models, sensors, and Internet of things devices in agriculture and described that, despite the existence of numerous agricultural models, their implementation in praxis is not satisfactory. This may be due to most models focusing on one subdomain, which will possibly make them less effective [16]. None of the evaluated research improved their sensors’ outcomes by combining their data with additional information [16]. Furthermore, the current perception of sensor benefit in agriculture does not justify the cost [16]. In contrast, ML algorithms may potentially be able to amplify the efficiency of these tools. Both Wolfert et al. [17] and Kamilaris et al. [18] stated that Big Data in Smart Farming is currently in the stage of early development. However, only two years later, Cabrera et al. [19] proposed their idea of the “dairy brain”, which would continuously apply ML algorithms to existing data commonly produced on dairy farms. These data originate from feeding schedules, herd management systems, and the milking parlor or automatic milking system software. Integrating different data streams would allow for improved management decisions [19,20]. Models like linear discriminant analysis or fuzzy logic can hereby detect events and initiate alarms [20].

### 3.2. Management

Farmers should be interested in their data and ML could aid their future management decisions. The three main drivers that motivate farmers to implement Big Data applications are as follows: 1) moving their business to the next level, 2) managing the farm as a system where data from subsystems can be used to improve the whole farm, and 3) enabling long-term and informed decision-making [21]. However, the infrastructure and tools to use such data are often still missing in the animal science community, and, therefore, large integrated datasets, which are essential for the development of reliable neural networks, are not available [22]. Farm management is possibly the primary source for data on most dairy farms. Many farmers hereby continuously produce semi-labeled data, which would be extremely valuable for the training of ML algorithms. An example is as follows: Reproduction and activity data give the farmer an indication on the ideal timing of insemination. The farmer inseminates the cow, documents this event in the herd management system, and, later on, labels the data by confirming both the conception and the calving date, and thus defines if the estrus detected by the monitoring system was true or false. Future prediction models could use this information to improve estrus detection. Thus, farm management data could be evaluated with a variety of concepts in mind, such as the classification of farms, estimation of energy and water requirements, or analysis of yield data.

#### 3.2.1. Classification of Farms

Machine learning, or more specifically, *k*-means clustering, allowed to discriminate different levels of farm mechanization by classifying conventional dairy farms [23]. This information is valuable for both commercial companies, which can use this information to address the farmers’ requirements and as such improve marketing activities, whereas federal offices can understand different levels of mechanization and use this information to improve political measures. Analogous work used cluster analysis (CA) to evaluate why farmers in Ireland were hesitant to adopt a spring rotation grazing planner [24]. Principal component analysis (PCA) and CA allowed for determining high adopters and low adopters and revealed that low adopters presented higher levels of constraint with specific regard to resource planning [24]. Such additional information allows better addressing communication and evaluating whether the farmers’ requirements are met by the applications offered [24].

#### 3.2.2. Prediction Models for Water and Electricity Consumption

Machine learning algorithms were able to improve the prediction of water and electricity consumption on pasture-based dairy farms [25]. The real-time prediction error was hereby enhanced by 54% for water (support vector machine) and 23% for electricity consumption (artificial neural network) compared with multiple linear regression models from previous studies [26]. This offered a tool for dairy farmers and policymakers that allows analyzing environmental factors of pasture-based dairy farming [25,26,27]. The previously developed support vector machine could predict the electricity consumption of grassland-based dairy farms with a relative error of 10.4% at the farm level and 5.0% across all farms included in the study [28]. The authors hereby also presented a practical approach of reducing energy demands by 4% when groundwater was used to precool the milk [28]. This shows how ML tools can quantify measures to improve efficiency, and therefore, aid the farmer in making informed management decisions.

#### 3.2.3. Performance Characteristics

Predicting the milk yield of individual dairy cows can help a farmer manage the herd more efficiently, for example, by supporting the early detection of diseases. Automatic milking robots and milking parlors offer continuous animal-specific data for this use case. Dynamic linear modeling was able to forecast the cows’ individual milk yields per milking from automatically collected milking robot data [29]. The study used a large dataset (*n* = 970,463 observations from 52 farms) of existing, automatically generated data to predict management relevant yields. The model further demonstrates the advantages of combined parameter evaluation, as both the somatic cell count (SCC) and the interaction between SCC and lactation stage affected yield prediction. Machine learning techniques also identified 15 variables from dairy herd improvement metrics that allowed to predict milk yield [30]. Artificial neural networks were hereby able to predict the first test day milk yield of heifers with a mean error below 4 kg. Furthermore, the authors showed a positive correlation between a high bodyweight and days in milk with first-day test milk, which exhibited a higher predicted milk yield [30].

Brotzman et al. [31] published a further prime example for the potential of ML approaches. The authors used PCA and CA to analyze dairy herd improvement data from 557 dairy herds with more than 200 cows each, including 319,934 cows and 22 variables (preselected from 992 measured variables). A PCA was applied to evaluate the 16 most important parameters from this dataset [31]. The average distance method for CA then allowed for an automatic classification of herd performance without a preconceived outcome [31]. Such a classification can help farmers to make informed feeding, culling, and breeding decisions as they learn more about the individual cows’ health, performance, and reproductive characteristics.

### 3.3. Physiology and Health

Sensors that monitor dairy cow physiology and health are commercially available [32,33]. However, due to a lack of publications on the performance of the underlying algorithms, it is not clear how well the decision support offered by these sensors currently performs. Regardless, scientists are now increasingly applying ML algorithms to use processed raw data from such sensors to develop decision support models.

#### 3.3.1. Body Condition Scoring

One way to observe the physiological state of cows is to monitor their body condition score (BCS). Advisors recommend regular BCS scoring to monitor both the individual cows and the herds’ health status [34]. The BCS (on a scale of 1–5 or 1–10) reflects the cows’ fat reserves, and can therefore indicate the requirement of changes in feeding or reproduction management [35,36]. Visual BCS scoring is time-consuming and requires expert training. Therefore, researchers aim to automate the scoring procedure. Machine vision has been used to automatically extract BCS via two-dimensional (2-D), three-dimensional (3-D), and thermal imaging, although these systems consider fewer body regions than are scored during direct observation BCS scoring [37,38,39]. Song et al. [34] addressed this issue and evaluated top-view images from two cameras that captured multiple areas of the cows’ body and successfully used the nearest neighbor classification model to classify BCS from an expanded selection of body regions. With this approach, the researchers achieved a classification sensitivity of 0.72 [34]. Furthermore, BCS was obtained from depth images using transfer learning where the best model achieved prediction accuracies of up to 96.82% (with a human error range of 0.5) [35]. However, larger and better distributed datasets are needed to evaluate the true prediction quality [35]. The application of DenseNet allowed for producing a model with fewer parameters, which performed better on BCSs below three [40]. Furthermore, adapting the AlexNet architecture to perform BCS and using a 19 layer deep convolutional neural network (VGG19 model) to classify the data resulted in a 67.39% success rate [41].

This set of research showed that ML methods can already be used to extract information from images and thus be employed within the management of dairy cows, for example, to adapt feeding strategies. However, the performance of such algorithms can still be improved. To achieve reliable ML predictions or classifications, decent quality of labeled data is important. It has recently been reported that the interobserver agreement of human BCS scorings (considered as the gold standard) is poor (concordance of correlation: 0.67), whereas the automatic system (BodyMatF, Ingenera, Switzerland) was more consistent in measuring the same score for a cow in another month than the human observer [42]. Therefore, future research needs to ensure high quality and consistency of their gold standard prior to the application of ML algorithms. In order to offer systematic decision support to the farmer, BCS data should be analyzed with feeding, milk yield, behavioral, or even meteorological data. It should be evaluated how these parameters can be best adjusted to improve the cows’ overall performance. An example: Environmental temperatures affect energy demand, which makes it sensible to adapt the feeding strategy to the climatic conditions. If this is not done, it is likely that the cow will react with lower milk yields, a drop in BCS or a change in behavior. This information could be implemented to create direct feedback to the farmer.

#### 3.3.2. Lameness

Lameness is one of the largest health-related issues in dairy farming, presenting one of the three main reasons for culling [43]. Therefore, researchers have attempted to detect lameness in dairy cows [44]. Most studies do not report on the efficiency of lameness detection, but rather give indications on behavioral changes, which makes it difficult to implement the findings in early warning systems [44], yet combining behavioral and gait parameters would offer the best approach for early lameness detection. Another approach is to fit deep learning algorithms, which detect lame dairy cows from video material [45]. This approach offers a low-cost and contactless alternative to sensors that need to be fixed to the animal [45]. The authors achieved a detection accuracy of 98.7% and a false positive rate of only 0.03. However, they only categorized “lame” and “not lame” cows in their dataset without specifying the score of lameness; the latter information is important for data interpretation, and as such, early detection [45]. Hudson et al. [46] looked at lameness from a different angle and used a dataset from 12,515 dairy cows in 39 herds to evaluate the effect of clinical lameness on reproductive success. Despite a discrete time survival analysis revealing a large correlation of the two parameters, a probabilistic sensitivity analysis exposed that the overall lameness occurrence would hardly affect the herds’ reproductive performance [46,47]. It is also possible to use standard management data to predict herds at risk of developing lameness [48]. A standard decision tree performed best in predicting herds at risk (sensitivity = 0.56, specificity = 0.89) [48]. Implementing herd management data for decision-making, without the requirement to apply additional sensors, would offer a great and low-cost opportunity to supply farmers with better management strategies.

#### 3.3.3. Heat Stress

Environmental factors, such as heat stress, can cause physiological changes in dairy cows that impact their affective state, biological functioning, as well as the natural living behavior [49]. Therefore, evaluating the effect of heat stress on cows presents another interesting application for the use of ML models. Although the relationships between heat stress and its physiological effects on dairy cows have been well documented, ML now allowed predicting and ranking physiological responses to environmental heat stress [13,49]. Nonlinear models (neural networks and random forest) hereby performed best in predicting the respiration rate, skin temperature, and vaginal temperature (R2: 0.61, 0.85, and 0.472, respectively) [13]. Furthermore, the ranking of environmental stressors showed that air temperature affected these physiological responses of dairy cows most, whereas wind speeds played a minor role [13]. These algorithms allow calculating thresholds of environmental parameters and can support farmers to decide when it is appropriate to implement heat stress reduction measures.

Cluster analysis further detected that physical activity of cows milked in automatic milking systems depended on temperature and humidity [50]. Physical activity was hereby lower in winter, whereas lower humidity levels increased the cows’ physical activity [50]. Future studies should include additional parameters reflecting the physiological state, yields, and interindividual interaction [50]. Furthermore, they should cover either a full lactation period or a full calendar year [50]. Genetic selection for high yielding dairy cows is linked to a lower tolerance to heat stress [51]. This in turn means that ML models could consider these aspects to ensure that genetic selection is not performed at the cost of tolerance to such stressful situations.

These findings are particularly interesting if we consider their significance in terms of being able to monitor any interference of environmental parameters during the analysis of studies with a different focus. As an example, this information could be used to analyze whether feeding strategies should be adapted to humidity levels, temperature, or even airspeed. It becomes evident that ML established first results that are not yet directly applicable in praxis, but researchers can now use these results in further analysis.

#### 3.3.4. Mastitis

Detecting diseases, such as mastitis, early would favor both economics and the cow’s welfare. Autoregressive integrated moving average models and CA were able to detect seasonal trends of mastitis pathogens in quarter milk samples, which are regularly assessed for microbial examination [52]. These classification models further detected that mastitis pathogens can be classified into both contagious and environmental categories, whereas previously, it has been reported that they could only be either one or the other [52]. The best marker for mastitis is the SCC. Being able to predict this marker from existing, automatically collected data, would aid the farmers decisions to prevent the onset of this particular disease. Data from a farm with 2,400 dairy cows and a total of 364,249 milking instances were recently analyzed to accurately predict SCC (percentage of accuracy: 84.9–82.23%) [53]. This data was automatically collected by an electronic inline monitoring system, where electronic conductivity, followed by lactose and fat content, had the largest weight in the prediction of subclinical mastitis [53]. The sensitivity rate for all tested models was over 93%, whereas the specificity was not satisfying, where naive Bayes had the highest specificity (39.7%) [53].

#### 3.3.5. Metabolic Status

Dairy cows are most susceptible to metabolic disorders, especially during early lactation. Detecting these conditions while they are only just developing would allow reacting before the disease could have a major effect, and thus reduce costs. Decision tree and random forest models were able to distinguish milk fever and displaced abomasum as the primary culling reason during the transition period of dairy cows in early lactation (up to 120 days in milk) [54]. Such algorithms could be implemented in practice to detect farm-specific risk factors [54].

Further, hyperketonemia is often used to detect poor metabolic adaptation syndrome in dairy cows, but it does not always prove reliable [55]. Therefore, Tremblay et al. [55] evaluated common metabolic health parameters with PCA and CA to define alternative separation values to better detect the onset of the syndrome. Future research should use this information to develop a prediction model for poor metabolic adaptation syndrome [55]. Cows’ metabolic health was also derived from cows’ plasma levels by applying CA [56]. In addition, it was possible to predict the cows’ metabolic status from standard farm data by applying random forest and support vector machines [56].

Attempts to detect subacute ruminal acidosis by analyzing behavioral anomalies with ML algorithms have failed [57]. Although it was possible to detect 83% of cases using *k*-nearest neighbor regression, the results were not useful in practice due to a false positive rate of 66% [57].

#### 3.3.6. Infectious Diseases and Spatial Analysis

Machine learning approaches can be useful to inform on the spread of infectious diseases. The *Mycobacterium* avium subspecies paratuberculosis causes paratuberculosis disease in dairy cows and presents a large economic threat to farmers [58]. In terms of infectious disease, research has particularly addressed the spread of infection. Clustering methods were able to detect seasonal clustering of *paratuberculosis* and revealed that animals born in summer were most likely to be infected by the highly contagious mycobacterium [59]. This information directly allows amending management decisions that minimize the risk of infection by timing calving accordingly. Further static and temporal networks were able to evaluate the role of direct and indirect contact networks in the spread of the disease [60]. This study was able to evaluate integrated datasets from the Italian national bovine database, as well as veterinarians’ farm visit data in a new context [60]. The analysis hereby revealed that the indirect spread of the disease by veterinarians visiting multiple farms played a much larger role than initially anticipated [60]. Geospatial mapping allowed to map hotspots of the disease outbreak [60]. The concept of spatial analysis is relatively new to precision livestock farming, although it has become part of daily life in other sectors. Google has implemented the utilization of Big Data; for instance, if increasing searches in Google address symptoms that include fever, cough, or illness, chances are high that flu season has started, and thus, it makes sense to advertise flu medication. Selemetas et al. [61] adapted this approach for risk mapping *Fasciola hepatica* infection in Irish dairy cows. Commonly known as liver flukes, *Fasciola hepatica* cause the parasitic worm infection fasciolosis [62]. Currently, the Ollerenshaw index, which considers a combination of rainfall, days of rainfall, and evapotranspiration, is used to predict *Fasciola hepatica* outbreaks in Ireland [62]. Milk samples from 500 farms were now combined with 108 variables covering environmental parameters such as soil, climate, and geospatial data to determine risk factors [62]. A random forest model showed that average rainfall was the most important predictor for the disease, followed by temperature, where temperature and rainfall were predictors in different constellations of means [61]. Furthermore, a strong prevalence of positive tests was detected in the southern regions of Ireland [61]. In this context, spatial analysis could offer a wide range of potential to limit the spread of diseases, including those transmitted by insects, such as blue tongue.

### 3.4. Reproduction

High-yielding dairy cows often come at the cost of poor fertility, leading to a greater number of calving to conception days, which is associated with high culling rates in Holstein Friesian dairy cows [2]. Recognizing estrus is vital for good conception rates, but this can be difficult, as only 50% of cows show the behavior “standing to be mounted”, which is considered the gold standard for estrus detection [63].

#### 3.4.1. Herd Management

Machine learning may be able to offer new approaches, as large labeled datasets are available at a transnational level through central herd management systems. Cook and Green [64] followed this approach and used a dataset from 8750 cows in 33 herds to perform multilevel logistic regression model analysis; they found that the conception rate depended on the cows’ production characteristics. Furthermore, the value of different multivariate models in terms of predicting conception rates were compared in Irish dairy cows [65]. Logistic regression models were the most promising to predict reproductive success for implementation in decision support systems [65,66].

Hudson et al. [67] stressed the importance of putting the results of data models into context, which is why they applied probabilistic sensitivity analysis to a dataset in which they investigated whether routine milk yield data could explain conception rates. Despite multiple studies describing a correlation between mastitis and reproductive success, these researchers found that this could hardly influence fertility rates at the herd level [47,67].

#### 3.4.2. Behaviors Associated with Reproduction

Aungier et al. [68] created activity clusters to examine the potential for estrus detection and successfully detected estrus in 90% of cows, while 10% of estrus events were missing and 17% were false positive. The authors described that their results were better than those of former studies as their dataset only included data from cows that were visually observed in standing estrus; therefore, further analysis would be necessary to determine the significance of activity clusters during estrus [68]. Abel et al. (2017) used a random forest classifier to identify lying, standing, walking, and mounting behavior in bulls on pasture from accelerometer data and found high correlations for lying and relatively high correlations for standing, walking, and mounting behavior compared with camera observations. The study shows that implementing ML can give further insight for reproduction management in bulls by providing additional information on the bulls’ behavior through automated monitoring. Commercial sensors currently available on the market as animal monitoring systems have already implemented some of these algorithms; however, the underlying algorithms have not been published. The advantage of successfully utilizing reproduction data would be twofold; improving conception rates can, first, reduce costs, and second, increase the longevity of cows. It is evident that, before becoming useful to farmers, these models need to be further developed.

#### 3.4.3. Genetic Selection

The first approaches of genetic selection evolved near the turn of the twentieth century. Since then, numerous statistical approaches were implemented to evaluate dam–sire performance [69]. Machine learning now offers the opportunity to predict outcomes from messy data even when explanatory variables are missing, yet at the disadvantage of performing as a “black box” [69]. Unbiased and reliable genomic predictions of Holstein sires’ lifetime merit were made by applying boosting algorithms from weak learners, where it was possible to predict genomic outcome [70]. Furthermore, if a PCA was used for variable selection and the genomic matrix was used as input, neural networks were able to predict milk yields (*r* = 0.67) [71]. Additionally, a random forest model was able to predict genomic regions that were associated with residual feed intake [72].

#### 3.4.4. Dystocia and Calving

Dystocia is a multifactorial problem in dairy cattle, especially at first calving. To respond to it appropriately, it is valuable to detect the condition early. Zaborski et al. [73] noted that ML offered new ways to detect dystocia and compared the performance of random forest and boosted tree models. Between the two models, boosted trees showed a higher sensitivity for detecting dystocia, but the specificity of the model was too poor to be used in praxis, as it would create too many false alarms [73]. Therefore, to date, we can see potential opportunities but cannot give reliable practical recommendations.

Calving prediction is an additional area where sensors can aid management decisions. Previous research has retrospectively been able to establish clear connections between animal behavior (rumination and lying bouts) and the onset of calving [74]. ML now offers the opportunity to predict calving events. Borchers et al. [75] used a variety of ML approaches to predict calving and found a high sensitivity and promising specificity. The authors used activity, lying behavior, and rumination time in neural networks and were able to create alerts 8 h before calving with a specificity and sensitivity of 80.4% and 82.8%, respectively. In contrast, the prediction of the 8-h period prior to calving was successful without including rumination data at specificity and sensitivity levels of 83.8% and 79.2%, respectively. Although this indicates good predictive values, the sample size included only n = 53 calving instances, thus, it would make sense to test the trained model on a larger dataset. Furthermore, Fenlon et al. [76] correctly predicted 75% of calving instances using a neural network and multinomial regression models, with 3.7% and 4.5% errors of the predicted probability, respectively. To date, some sensors that promise early warnings for calving detection are available on the market; however, little research defining the reliability of such alarms is available. The reviewed studies indicated that the prediction of both estrus and calving can be improved through ML algorithms. However, the prediction outcome is not yet satisfactory. The field of anomaly detection offers a wide range of potential for future research. Particularly, the combination of different parameters could add new insights and may increase the reliability of predictions.

### 3.5. Behavior Analysis

The affective state of animals has recently gained attention, as it could be the key to assess animal welfare [77]. Although numerous methods for the assessment of the affective state, or feelings, of the animals are available, they are at risk of producing false positive results [77]. Behavioral analysis provides direct feedback on animals’ physiological state; therefore, sensors that measure these behaviors have become available.

#### 3.5.1. Sensor-Based Behavior Classification

Sensors in dairy farming are often based on the classification of accelerometer data. The ultimate idea bringing value to these sensors is providing decision support to the farmer. In 2013, Rutten et al. [33] reviewed sensors in dairy farming and reported that although a number of sensors were available to monitor the behavior of dairy cows, none of them were able to provide decision support for the farmers. Since then, the systems have evolved. Machine learning now offers the opportunity to utilize additional, more indirect data sources. However, only little is known about the quality of decision support systems in praxis. Determining behavior during grazing, for example, has been highly challenging due to connectivity issues that make it difficult and costly to install sensors and receive their information outside of the barn [32]. Behavioral models were now able to distinguish between grazing, walking, and resting behavior from global positioning system (GPS) data in pasture-based dairy cows through temporal positioning [78]. All classifiers hereby distinguished walking with a classification accuracy of 0.94 or more [78]. JRip, J48, and random forest classified resting with an accuracy of 0.85 or more, whereas all models classified grazing behavior rather poorly (accuracy: 0.16–0.72). The best classifier, JRip, reached a weighted average classification accuracy of 0.85 with a false positive rate of 0.1 [78]. This research was continued to predict cow behavior from GPS locations at a 5-s logging frequency and successfully identified the change points from the behaviors of walking, grazing, and resting (for walking and standing, 90.2% of change points were identified within 4.45 min of the true changepoint) [79]. This analysis was performed by applying the R package “changepoint” [79,80]. The application can monitor animal behavior with a very low sampling frequency, allowing for prolonged battery life, although the monitoring time still indicates potential for improvement.

As proposed in previous research, a team of scientists recently applied ML algorithms to improve behavior analysis based on data from an accelerometer that was mounted to the cow’s neck and leg [81]. When the accelerometer was mounted on the neck, they achieved good results for the classification of feeding behavior (95–98% sensitivity and 88–92% specificity), but when it was mounted on the leg, they reached good classifications of lying behavior (sensitivity and specificity > 93%) [81]. Support vector machines performed better than the other tested algorithms and are additionally advantageous in that they require few computing resources with reduced energy needs after the model training is complete [81]. A good prediction of both behaviors was achieved using data from both the neck- and leg-mounted sensor [81]. Data from radiofrequency identification (RFID) sensors, which are implemented in dairy farms with automatic milking systems in a standard capacity, were employed within a neural network to track cows [82]. The authors tracked cows for up to 20 min after passing the RFID recognition [82]. Although this approach shows some potential, further work remains before we can conclude the practicality of use in a standard application.

The analysis of social networks also became possible through ML methods. Boyland et al. [83] applied a number of ML approaches, including supervised learning and clustering, to examine the social structures of dairy cows in commercial housing systems from proximity logger data. They found that animals only formed clusters within the specific animal and showed only little social stability within the herd structure. Contrarily, Foris et al. [84] demonstrated that cows formed relatively stable contact networks, and that these contact networks can influence the individual cows reactions within the group [85]. ML could offer the opportunity to continuously monitor the stability of such groups and their effect on affective state or physiological parameters.

#### 3.5.2. Vision-Based Behavior Monitoring

Analyzing behavior and social networks from video material would offer the advantage that it is not required to install sensors to the animal. Vision-based analysis of dairy cows has proven challenging in the past due to the difficulties of identifying the individual cow. Shen et al. [86] recently addressed this issue by applying convolutional neural networks and implementing the Yolo model, as well as the AlexNet model for individual cow identification. Cows were identified with an accuracy of 96.65% [86]. However, it must be pointed out that these algorithms only work well for colored cows and have problems identifying black cows [86]. This is also a problem in the identification of other breeds with uniform markings and colors or both, such as Aubrac, Grey, Angus, Limousine, or Brown Swiss cattle. Jiang et al. [87] further classified cows’ heads, backs, and legs from images by training a FLYOLOv3 model; they achieved an accuracy of 99.18%, a recall rate of 97.51%, and an average precision of 93.73%. Particularly, the combination of animal identification with additional algorithms, such as the combination with BCS, offers novel ways of using physiological and behavioral traits for management decisions [40]. The capabilities of neural networks are expanding rapidly, as shown by Salau et al. [88] that were able to implement social network analysis from dairy cows by analyzing video data from multiple cameras. To date, the authors had to mark the cows by hand, but this could be solved through improved neural networks in the future. Information on social networks in dairy cows could not only allow learning more about their social structures and behaviors but may also allow advancing development of dairy housing systems; it could further be used to detect abnormalities in behavior that could be integrated into decision support systems.

Guo et al. [89] recently developed a machine vision model for the recognition of calf behavior by combining background subtraction and inter-frame difference models. They managed to distinguish behaviors of calves housed in igloos with detection rates of over 90% (pen entering: 94.38%, pen leaving: 92.86%, standing or laying in a static position: 96.85%, and turning: 93.51%), as well as feeding and drinking behaviors, at near 80% (79.69% and 81.73%, respectively) [89]. Transferring this study to a loose housing dairy barn would remain challenging, as it requires the installation and combined evaluation of multiple cameras within the barn.

#### 3.5.3. Anomaly Detection

Measuring behavior of cows offers a great opportunity to quantify the animals’ normal behavior, and therefore, detect anomalies of this behavior when the animal has altered its behavior due to a health issue. Machine learning methods can predict the normal behavior of the animal and create an alert when actual and predicted behaviors differ from a defined threshold. A variety of systems using this approach are already implemented in commercial products. The Data Driven Dairy Decisions for Farmers (4d4f) framework created an overview of sensors currently available to monitor animal behavior [4]. For instance, Smartbow is an eartag-based accelerometer that detects anomalies of rumination behavior and activity levels that inform the farmer about problems or detect estrus, as well as locate animals in the barn [6], while the SmaxTec Rumen PH bolus can be used to detect ruminal acidosis [7]. False positive alerts are a significant issue for some systems, as too many such alerts will be annoying to the farmer in praxis.

#### 3.5.4. Behavior Related to Metabolic Status

Behavior monitoring offers additional fields of application as the cows’ behavior gives indications of their physiological state. Farmers could use this information to detect and react to physiological changes. A modification in feeding behavior, for example, can indicate the onset of health disorders at an early stage [90]. González et al. [90] detected 80% of acute health disorders in dairy cows one day before their diagnosis by applying an algorithm that creates an alert if a cow’s feeding behavior drops below its seven-day average minus 2.5 standard deviations [90]. Particularly, rumination and feeding behavior are possible indicators for improving dairy cow management. Wagner et al. [57] evaluated whether ML algorithms could be useful to predict subacute ruminal acidosis from positioning data that reflect cows’ activity. They reported that, among the tested ML algorithms, *k*-nearest neighbor performed best, with 83% true positives; unfortunately, the false positive alert rate was 66%. This shows that it is still necessary to solve these issues for anomaly detection.

Data acquisition of animal behavior in research trials is often linked to installing sensors on the animal, followed by a data export and analysis procedure, making it difficult to actually use these data for early warning systems. Ultimately, the investigated studies showed that the predictions of metabolic health exhibit high potential but are currently not satisfactory for implementation in practice. This raises questions about commercially available products that use, but have not published studies evaluating the performance of such algorithms.

### 3.6. Feeding

Precision feeding of dairy cows presents an opportunity to improve the herds’ efficiency as the feeding regime directly affects the cows’ milk yield [91]. Therefore, animal-specific feeding regimes have been discussed, where intake predictions have become more relevant. Dorea et al. [92] predicted dry matter intake of silage in dairy cows using milk spectral data from infrared spectroscopy and applying partial least squares and artificial neural networks. However, adapting the feeding method did not affect the performance of high-yielding dairy cows in recent research, indicating that concentrate use could be handled more efficiently [93,94].

#### 3.6.1. Group Feeding

Cluster analysis can improve the analysis of animal-specific data. Cluster graph models were successfully used on time series data of cows milked in automatic milking systems to categorize herd characteristics and classify cows based on five different parameters (number of daily milking procedures, parity, average daily activity, milking regularity, and cow body weight) [95]. To analyze behavioral and production features, *k*-means clustering models were implemented for each of these parameters [95]. This information can be processed to automatically group the animals into individual feeding groups.

The idea of using existing farm data—more specifically, herd management, milking system, genetic and genomic, monthly milk testing, feed, and milk processor data—for real-time continuous decision making was recently introduced [19]. Machine learning methods were applied to large datasets, successfully deriving nutritional groups, detecting cows at risk of clinical mastitis, as well as continuously predicting the onset of clinical mastitis, with relatively high levels of sensitivity and specificity [19]. Knowing and implementing this information can aid to improve both the health of the individual cow and the entire herds’ fitness.

Glatz-Hoppe et al. [96] further used linear regression models to evaluate a dataset consisting of 7.3 million milk recordings and were able to show that traditional feeding strategies were not ideal. The data showed that, based on a breed-specific threshold of fat:protein ratio and a protein minimum value, it was possible to estimate a lack of energy and thus estimate the risk of ketosis [96]. This shows how large datasets that are produced irrespectively of experimental research questions can create immense advances in informed feeding strategies. Evaluating such datasets with predictive ML models could allow integrating further parameters, such as behavior or climatic conditions, to create an even more advanced and adapted feeding regime.

#### 3.6.2. Grazing

Consumers’ demand for grazing dairy cows increased with their awareness of animal welfare; research has correspondingly aimed to improve the efficiency of grazing dairy systems by implementing sensor technologies. Shalloo et al. [32] stressed that grassland-based dairy systems have economic advantages, as the direct utilization of grassland strongly reduces production costs. This hypothesis is supported by Gazzarin et al. [97] that reported a significantly higher income in grazing systems through a noteworthy reduction of labor costs. Grazing systems are further beneficial, due to calving, and thus peak energy demands being better synchronized with peak grass growth [98]. Efficient grass utilization and cow health, and within cow health fertility, are the most important drivers of efficiently managed pasture systems [32]. Those sensors monitoring the animals physiological state should be combined with sensors that measure biomass, as their interaction could aid informed pasture management decisions [32].

This approach has been implemented, where feeding time correlated with feed intake, which can be useful for estimating intake rates on pasture [99]. Hills et al. [100] also describe that sensors can potentially give indications to optimize nutritional demands through individualized feeding strategies, and thus improve the management of grazing cows. However, finding the right parameter or the right combination of parameters remains challenging [100]. Shafiullah et al. [101] have taken their research a step further and were able to detect herbage shortages in the feeding and activity behaviors of grazing dairy cows and found that rumination chews per day and grazing bites per minute were the best predictors for insufficient grazing [101]. Machine learning models (support vector machine, random forest, and extreme gradient boosting) hereby performed better than the general linear model did in cross-validation [101]. To evaluate their potential for practice, it is necessary to validate these ML models with novel data.

### 3.7. Constraints of Data Availability

Beyond the capability of ML algorithms, the availability of large datasets plays a vital role in enhancing data-driven management decisions, as ML approaches depend on large, high-quality datasets, yet data availability is poor. Wolfert et al. [17] described that stakeholder networks are organized into two particular extreme scenarios. Either the networks use open-source solutions to keep their resources open and allow for interoperability of data or they are closed and proprietary. Most sensors or sensor systems currently aim to fit one specific purpose, implying that they are manufacturer-specific, and as such, encourage vendor lock-in. Linking different data sources from different farms would offer the most promising potential to develop better algorithms that could improve farm management [102]. Unfortunately, the data are difficult to integrate with additional data sources as companies restrict access to their records [103]. This not only leads to farmers having to input the same data in multiple software programs but also prevents linking the data to optimize dairy cow management on a multifactorial level. Research has introduced the idea of integrating data streams, highlighting the potential of integrating general farm data to improve automated monitoring of dairy cows over 10 years ago, when researchers implemented a fuzzy logic approach for abnormality detection [20], yet with current issues on data restrictions and availability, it is difficult to move forward. Equally, when applying ML algorithms in the farming environment, we must keep in mind specific issues, such as cybersecurity and defense mechanisms [104].

### 3.8. Robustness of Models, Cross Validation, and the Risks of Machine Learning in Dairy Science 

With the powerful outcomes of ML algorithms in mind, it is essential to understand the underlying algorithms, and is therefore necessary to properly split training and testing datasets, tune parameters appropriately, avoid overfitting, and ensure that the conclusions drawn are realistic [105].

If dairy scientists apply ML techniques, we face the difficulty that not everyone has acquired the same level of understanding of the underlying algorithms. The scientific peer review systems ensures quality through expert evaluation [106]. As ML methods are now increasingly being applied it may be difficult for some supervising bodies, or reviewers, to evaluate the quality and scientific integrity of ML studies. This can develop into a potential pitfall for young scientists, their supervisors, the reviewers, and, therefore, the scientific peer review system. Therefore, it is valuable to point to a few important studies, which demonstrate critical aspects in evaluating the performance of ML research.

Flach [107] points out that the performance of ML models can be measured by indicators such as accuracy, true and false positive rate, precision and recall F-score, Area Under the Curve, and Brier score. Each of these evaluation indicators serves a different use case where it is considered bad practice to over-report [107]. The author further stresses the need for a responsible performance evaluation in ML and describes good and bad practices with a suggested way forward [107]. Particularly important aspects from this study: 1. Researchers should set a clear objective for their study and from this perspective justify the chosen evaluation method. 2. There is a need for a measurement theory of ML models, of which there is currently much less knowledge than on ML technology itself [107].

Additionally, it is particularly important to test the robustness of ML models, because they are at risk of overfitting, where the model is not only fitted to the data, but also to the underlying noise [108]. It is therefore necessary to test the validity of the trained model, to evaluate its true performance on novel data [108]. One way to do this is by splitting the data into training (70%) and testing (30%) datasets or with larger datasets into training 60%, testing 20%, and validation 20% [108]. One split of the dataset is used to train model, while the other split is used to test the model on a novel testing dataset. Splitting the datasets into three folds results in smaller datasets, which makes it more feasible to use cross-validation in the case of small datasets [108]. In general, it is vital to recognize scientific errors and so ensure the quality of research developed with these methods.

Within peer-reviewed papers, authors are often responsible for a specific topic within the paper. For example, a data scientist or statistician may be responsible for the data analysis, whereas the dairy scientist will cover the aspects of dairy science. The reviewers within the peer review systems are however expected to be experts in both, despite perhaps now being familiar with the methodology. To prevent this from becoming a problem, it might make sense to reorganize the peer review strategy, where reviewers are only responsible for reviewing the part of the paper, which addresses their specific area of expertise.

### 3.9. Synthesis

ML algorithms have become common research tools in dairy science, and they can advance knowledge, particularly in areas where predictions are required. Although traditional statistical methods in the dairy sector evolved an inevitable foundation of information, ML algorithms provide new opportunities for further advanced data-driven discoveries. The studies in this review show that ML is commonly applied in most sectors of dairy science, such as reproductive and feeding management, BCS scoring, health monitoring, and behavioral analysis. However, despite ML having become a substantial part of data analysis, the advantages of these algorithms have not managed to solve the open issues.

Currently, the average age of Holstein dairy cows is 3.3 years, with 2.45 lactations [109]. To improve this situation, integrating various data sources could offer new approaches to farm management. To be more specific, we recognize that estrus is detectable through increased activity levels, but we also know that activity levels increase with a change in herd structure or drop with rising humidity levels. Therefore, in the future, the thresholds for estrus detections could be adapted to the baseline behavior recovered from integrated data to improve the accuracy of estrus prediction and decrease false positive results.

ML algorithms could improve feeding strategies. To date, farmers feed cows according to lactation curves, primarily focusing on the lactation stage and milk yields. However, the scientific community acknowledges that the feed content and structural composition can affect both the behavior of the cow and their individual daily milk yields. In addition, feed advisors recommend feeding cows according to their BCS. Linking these various data sources and analyzing them could promote new strategies for animal individual feeding that may even continuously account for the cows’ physical state.

This raises the question of why ML methods are not being fully exploited in dairy farming today. One reason could be the lack of availability of multiparameter datasets. Well-described, multifactorial, high-quality, and freely accessible datasets would allow for development of better algorithms and possibly reduce false positive alerts in monitoring systems. To address this, data on animal-specific behavior and yields from a variety of farms would be particularly interesting. However, smaller animal-specific datasets could also be interesting for specific research questions. Although sensors that continuously collect data are available, hardly any will provide their data in a useful manner to create a platform between sensors, allowing the consideration of multiple parameters for advanced prognosis, categorization, or anomaly detection. Thus, two recently initiated projects, “Dairy Brain” and “Smart Dairy Tracer,” have addressed multiparameter continuous data analysis and are currently working toward demonstrating the chances of integrated datasets [110,111].

## 4. Conclusions

The reviewed literature shows that wide varieties of parameters determine the performance and health of dairy cows, and these must be managed appropriately to improve the efficiency of dairy farms. Therefore, multiple data sources need to be interlinked. This often fails due to the restricted availability of public datasets, commercial sensors not providing standardized data infrastructure, and vendor lock-in. Improving these data sources is essential to aid the development of data-driven dairy farm management and would allow developing reliable and multifactorial models, which could provide better guidance on the appropriate management of feeding, reproduction, health, milking, or resources. In addition, we conclude that many researchers have recognized the potential of ML, and it is now time to start implementing these powerful tools in multidisciplinary collaborations between dairy and data scientists, to realize their potential impact.

## Figures and Tables

**Table 1:** Search strings and results of peer-reviewed papers (articles/reviews) in Scopus and of all articles in Google Scholar since 2015; searches last performed on 24.06.2020 and 25.06.2020 in Scopus and Google Scholar, respectively. The indents in the column “search string” indicate the hierarchical structure of the search strings.

	Google Scholar			Scopus	
Search String	In Title	In Document	Article Title	Abstract Title Keywords	In Document
Machine learning agriculture	81	39,100	17 (13/4)	869 (808/61)	18,453 (17,133/1320)
Machine learning dairy	33	15,900	19 (19/0)	109 (102/7)	2192 (1861/331)
Random forest dairy	3	15,900	3 (3/0)	46 (46/0)	1055 (930/125)
Cluster* dairy	41	18,200	36 (35/1)	1174 (1143/31)	14,714 (12,075/2009)
Neural networks dairy	12	15,800	12 (12/0)	112 (108/4)	4,133 (3368/765)
Deep learning dairy	9	15,900	5 (5/0)	25 (21/4)	863 (654/209)
K-Nearest neighbor dairy	0	379	0	10 (10/0)	123 (114/9)
Bayesian models dairy	1	12,600	4 (4/0)	213 (209/4)	4318 (3920/398)
Support vector dairy	2	16,800	2 (2/0)	51 (49/2)	1803 (1464/339)
Decision tree dairy	3	16,600	3 (3/0)	68 (67/1)	1449 (1222/227)
Ensemble learning dairy	0	4010	0	13 (12/1)	298 (249/49)
